# Reinventing Patient Support and Continuity of Care Using Innovative Physician-staffed Hotline: More than 60,000 Patients Served Across 15 Medical and Surgical Specialties During the First Wave of COVID-19 Lockdown in Qatar

**DOI:** 10.1007/s10916-023-01973-w

**Published:** 2023-07-19

**Authors:** Mohamed Arafa, Walid El Ansari, Fadi Qasem, Abdulla Al Ansari, Mohammed Al Ateeq Al Dosari, Khalid Mukhtar, Mohamed Ali Alhabash, Khalid Awad, Khalid Al Rumaihi

**Affiliations:** 1 Urology Department, Hamad Medical Corporation, Doha, Qatar; 2 Andrology Department, Cairo University, Cairo, Egypt; 3 Weill Cornell Medicine – Qatar, Doha, Qatar; 4 Department of Surgery, Hamad Medical Corporation, Doha, Qatar; 5 College of Medicine, Qatar University, Doha, Qatar; 6 Department of Orthopedics, Hamad Medical Corporation, Doha, Qatar

**Keywords:** Hotline, Telemedicine, Telehealth, Triage, Virtual care, Quality of health care, Innovation, Helpline, Utilization

## Abstract

Rising disease prevalence early during the COVID-19 pandemic in the State of Qatar led to stoppage of all non-emergency health care services. To maintain continuity of care and information exchanges for non-emergency patients, a physician-operated telephone hotline was set up that involved triage followed by immediate consultation with a specialized physician. We describe the initiation and evaluate the operations of the Urgent Consultation Centre (UCC) hotline manned by 150 physicians and aimed at urgent non-life-threatening consultations at Hamad Medical Corporation, the public health provider in Qatar. UCC established a hotline to triage inbound patient calls related to 15 medical and surgical specialties. For calls between April-August 2020, we describe call volume, distribution by specialty, outcomes, performance of UCC team, as well as demographics of callers. During the study period, UCC received 60229 calls (average 394 calls/day) from Qatari nationals (38%) and expatriates (62%). Maximum total daily calls peaked at 1670 calls on June 14, 2020. Call volumes were the highest from 9 AM to 2 PM. Response rate varied from 89% to 100%. After an initial telephone triage, calls were most often related to and thus directed to internal medicine (24.61%) and geriatrics (11.97%), while the least percentage of calls were for pain management and oncology/hematology (around 2% for each). By outcome of consultation, repeat prescriptions were provided for 60% of calls, new prescriptions (15%), while referrals were to outpatient department (17%), emergency department/pediatric emergency center (5%), and primary health care centres (3%). We conclude that during a pandemic, physician-staffed telephone hotline is feasible and can be employed in innovative ways to conserve medical resources, maintain continuity of care, and serve patients requiring urgent care.

## Introduction

With the declaration of the COVID-19 pandemic, ways of taking care of one’s health and the relationships with a physician changed [1]. To lower the number of confirmed cases and reduce pressure on hospitals, the lockdown went into effect [2]. As most countries implemented the lockdown, providers across all specialties rapidly embraced the use of telehealth/ telemedicine [3] to maintain the continuity of health services and avoid missing emergency conditions, while minimizing face-to-face visits [4]. Such move from traditional care to virtual health became critical to protect patients, clinicians and the community from exposure, whilst providing a platform for providers and patients to interact at any time [5, 6]. In response, in the State of Qatar, with the start of the pandemic, Hamad Medical Corporation, the public tertiary care provider, initiated a dedicated Urgent Consultation Center (UCC) hotline to triage, consult, identify diseases, and treat /or refer according to the condition’s urgency [7].

Research has recognized the effectiveness of hotlines. For example, telephone delivered psychotherapy was similar to traditional face-to-face therapy [8], and hotlines provided specialized mental health support [9]. Hotlines overcome geographical isolation [10], stigmatization, transportation [11], reduce the pressure of consultation and provide a safe environment to express emotions [12]. Unsurprisingly, hotlines are a very popular crisis-intervention measure globally [13].

Telephone helplines are well-established conduits for mental health protection and suicide prevention, offering immediate, anonymous, cheap and accessible support [14]. Likewise, hotlines provide information about resources e.g., emergency shelter, emotional support, and help in safety planning [15]. In Egypt, calling the hotline was one of the most frequent practices to deal with COVID-19 symptoms [16]. In New York, a hotline was chosen over formal types of virtual medical appointments (e.g., telehealth) as it was widely accessible (anyone could call regardless of literacy level/ socioeconomic status); there was no cost, registration, appointment, or insurance verification, making it easy for patients and easing the burden on emergency medical services; and relying on a telephone eliminated technology barriers and facilitated clinician participation on short notice [17]. Hence, due to the unique characteristic of the epidemic, hotlines have become the most convenient way of rescue.

During COVID-19, hotline services were implemented in many countries [2, 18–29]. Likewise, hotline services spanned many individual healthcare specialties, providing support/health information for conditions including COVID-19 [17, 30], suicide prevention [13], dermatology [31], mental health [21, 29, 32], sexual assault [33], dialysis/transplant [18], child abuse/neglect [34], or dental care [4, 6].

Notwithstanding, the literature reveals knowledge gaps. First, most studies reported hotline service/s operated by non-physicians. These included triage nurses [35], nurse specialists [31], trained volunteers [13], trained volunteers supervised by health professionals [36], counselors [2, 37], psychotherapeutically trained counselors [38], psychologists/clinical psychologists with psychotherapist qualifications [32], law enforcement/social service agencies [15], or operators [39]. In China, most of the 36 psychological assistance hotlines during the COVID-19 epidemic were manned by counselors with some qualifications, certified by different institutions, received different training and supervision, and their experience varied widely [40].

Second, clinician-staffed hotlines are rare, and when reported, such hotlines were limited to COVID-19 support. In New York, a clinician-staffed COVID-19 hotline was established, but no details were provided on who such staff were [17]. Similarly, a physician-staffed COVID-19 hotline included social care referrals for patients requiring to self-isolate [30].

Third, most hotlines were established for a single condition, rather than the broader spectrum of medical and surgical specialties. e.g., abortion [41], psychological support [38, 39], opioid use disorder [42], combating COVID-19 [43], domestic violence [44], inflammatory bowel disease [45], or respiratory support [46].

Therefore, to bridge these knolwedge gaps, the current service evaluation describes the initiation and inauguration of a UCC hotline manned by physicians to triage and respond to all medical and surgical specialties during the first five months of the COVID-19 lockdown in Qatar. We analyzed the caller, call and specialty characteristics, as well as call outcomes of all hotline calls across 15 medical/surgical specialties.

## Materials and Methods

### Ethics, Design, and Participants

Our hospital institutional review board provided approval for this service evaluation. It is a retrospective analysis of data routinely collected as a component of service evaluation/ audit. A total of 77,217 calls were received. We excluded COVID-19-related/general information queries (N = 16,988, 22%), leaving 60,229 calls that are included in this analysis.

### Setting

#### Healthcare Landscape in Qatar

The Ministry of Public Heath (MOPH) is the supreme healthcare authority in Qatar. Hamad Medical Corporation (HMC) is the largest public healthcare provider and oversees the 12 public hospitals. There are 27 primary health care centers (PHCC) in Qatar, and many private clinics and hospitals.

By mid-March 2020, in response to COVID-19 pandemic, Qatar implemented restrictions to limit viral transmission. For public healthcare, all outpatient visits were converted to telehealth. Physicians called the registered patients at the time of their appointment. Elective surgeries, except oncology-related, were stopped; and emergency departments (ED) were limited to life-threatening conditions. Many public hospitals/PHCC were converted to COVID-19 facilities. Likewise, all private clinics were temporarily closed and most private hospitals halted their activities.

These restrictive measures, however, resulted in an increased likelihood of disruption of continuity of care for some patients with urgent non-emergency conditions e.g., (1) patients with new non-COVID-19 complaints with no hospital appointment; (2) chronic patients experiencing changes in co/morbidities with no hospital appointment at a near date; (3) patients developing side effects to treatment/s; (4) chronic patients who missed their outpatient department (OPD) appointments and needed medication refill/s; and, (5) visitors in Qatar with no access to public healthcare. Before the pandemic, such patient groups could use public healthcare as walk-in, or use private healthcare.

### Procedures

#### Creation of the Urgent Consultation Center (UCC)

HMC recognized this risk of disruption and the need for a healthcare hotline, the UCC, to: (1) provide safe care access for patients with urgent non-life-threatening conditions; (2) identify high-risk patients needing emergency intervention/s but afraid of infection; and, (3) avoid unnecessary hospitals visits to reduce the risk of COVID-19 transmission to healthcare providers/patients. A steering committee was assigned to create the UCC and necessitated several steps, as outlined below.

##### Workspace

Workspace (300 m^2^) in an HMC administrative building, purposively chosen away from the patient areas, was allocated to accommodate the triage/specialty physicians of all medical/surgical specialties. The workspace was in accordance with COVID-19 regulations: each area staffed with a maximum of 8 persons, at least 2 m apart; fiberglass sheets separated the desks; COVID-19 screening and temperature measuring were undertaken on entry to office areas before every shift; personal protection equipment, sanitizers and hand wash were used; thorough regular sanitization of the desk areas and computers etc. was undertaken; and, staff stopped using the biometry attendance machines.

##### Communication/Information Technology (IT)

The Ministry of Communications provided a hotline number for UCC. HMC’s IT department installed computers with access to the hospitals’ patient hospital information system (HIS), and connected the landline telephones.

##### Staffing

Fifteen triage physicians were hired and trained on the use of hospital technology (operation system, Cisco phones, etc.), creation of UCC encounter, telehealth communication skills, and hospital policies (patient rights/ privacy). In addition, each HMC department assigned attending senior physicians (consultants/specialists) to the UCC, chosen for their ability to assess the urgency of the calls, and appropriately serve patients through teleconsultation.

##### Advertising/Promotion

HMC’s Communications Department initiated a campaign across media outlets (newspapers, television, radio) and social media platforms to popularize UCC, orienting the general public about the service.

#### Workflow at UCC

**Fig. 1:**
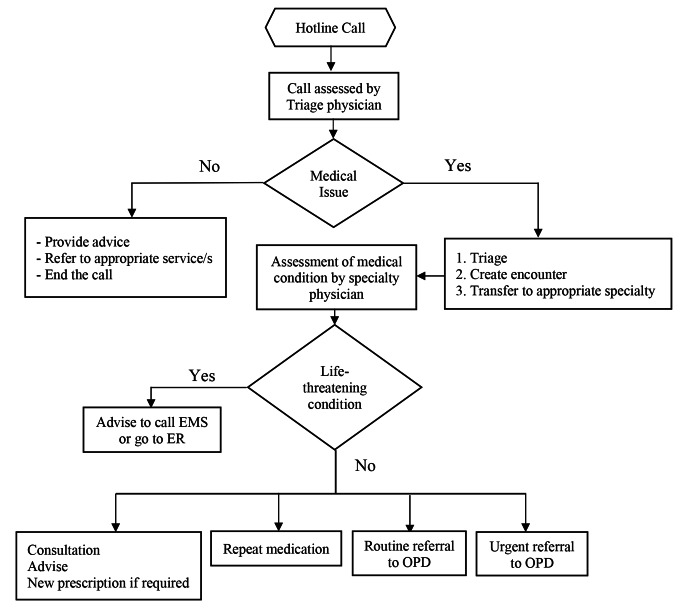
Workflow chart. EMS: emergency medical services; ER: emergency room; OPD outpatient department

**Fig. 2:**
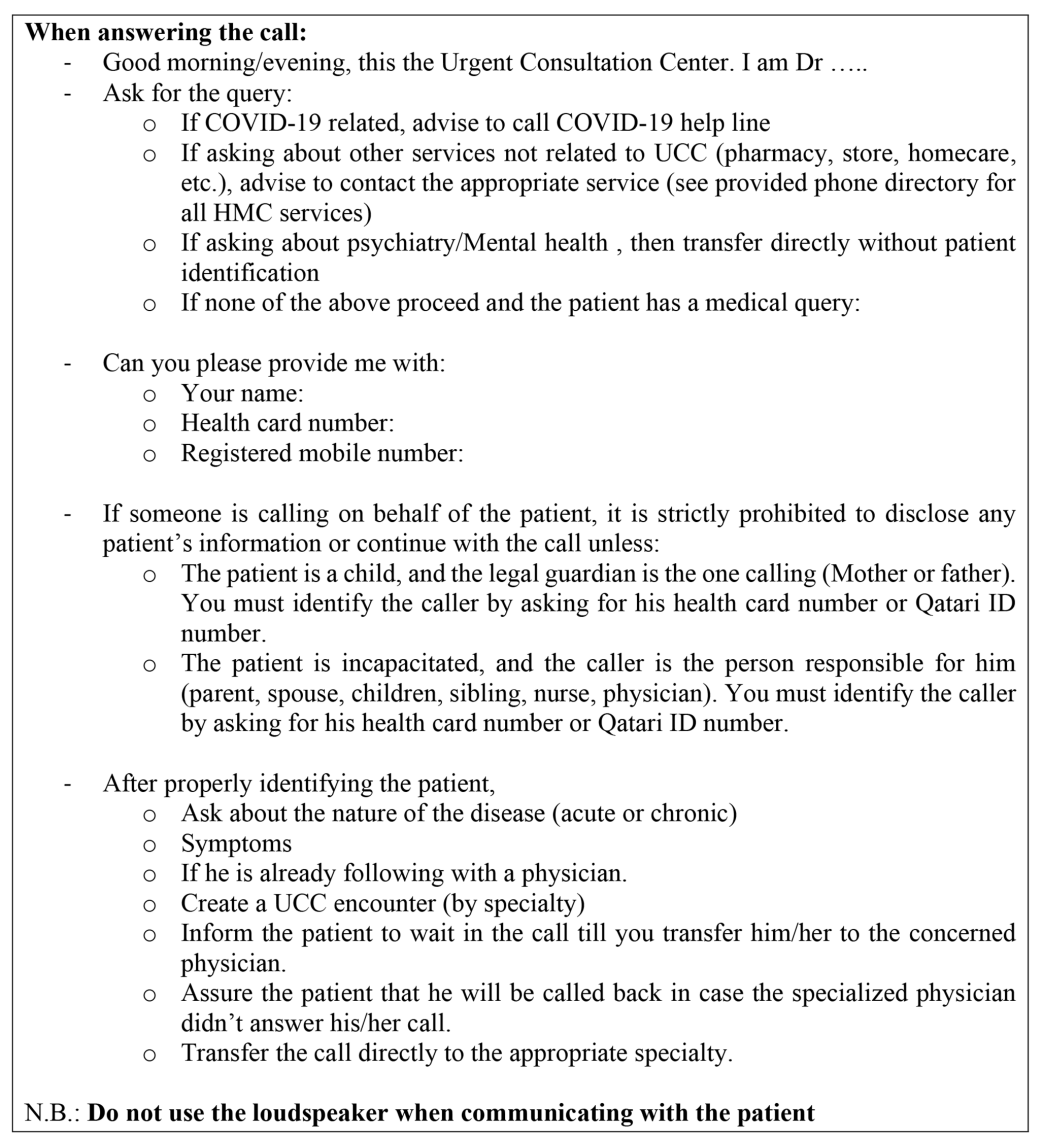
Triage Physicians Workflow Protocol. UCC: urgent consultation center; EMS: emergency medical services; ED/PEC: emergency department/ pediatric emergency center

Patients would call the tollfree hotline (Fig. 1). Triage doctors attended the call, identified the patient, and carefully assessed the query/complaints using the triage physician protocol (Fig. 2). Calls were then directly connected to the relevant specialty physician. Triage physicians managed general guidance calls and referred COVID-19-related calls to the MOPH/COVID-19 helpline.

**Fig. 3:**
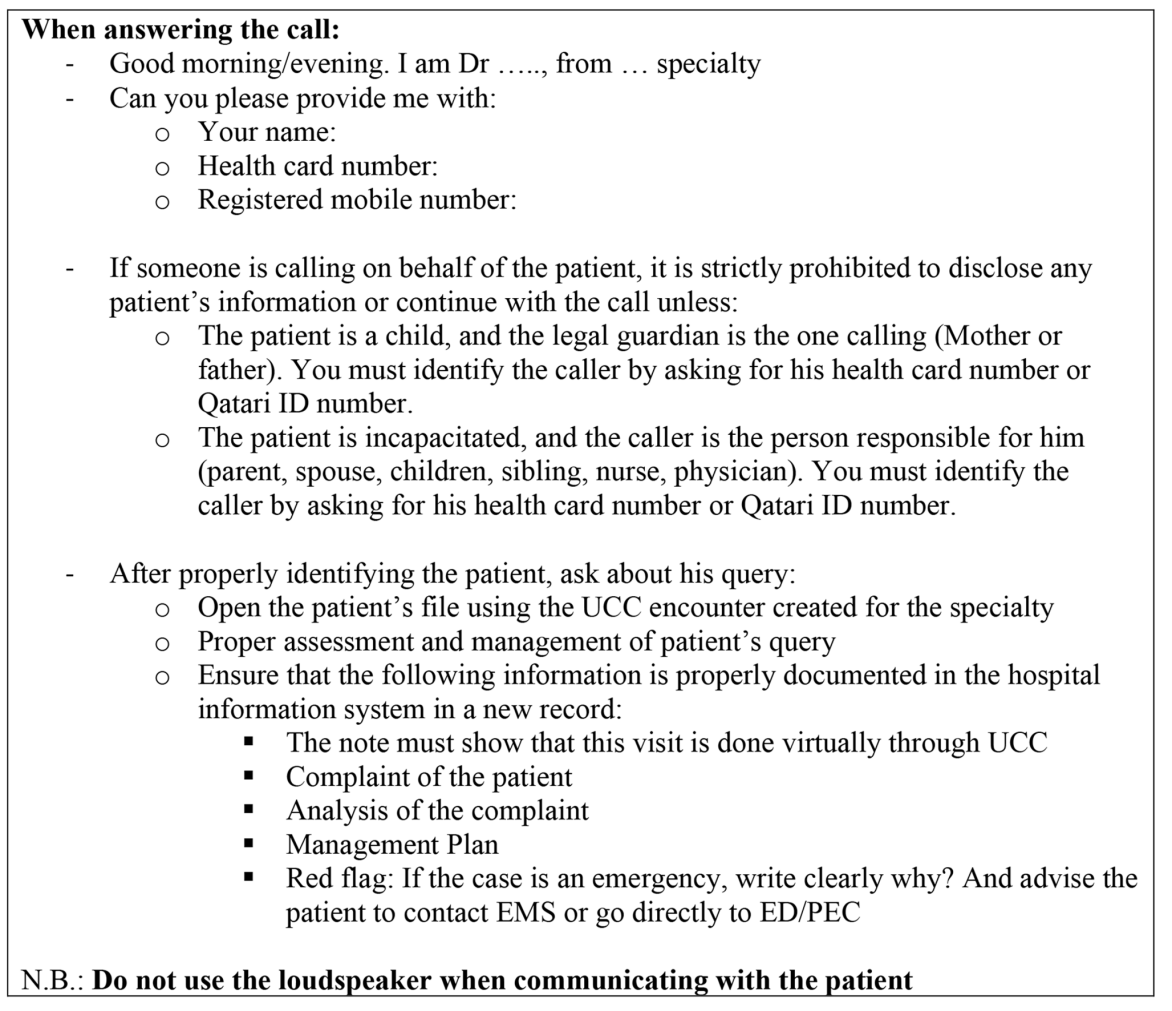
Specialty Physicians Workflow Protocol. UCC: urgent consultation center; EMS: emergency medical services; ED/PEC: emergency department/ pediatric emergency center

Specialty physicians then assessed the patient’s condition, managed accordingly based on the specialty physician protocol, and documented the information in the hospital HIS (Fig. 3). Life-threatening or suspected emergency cases were guided to call emergency medical services (EMS) or go to the ED/Pediatric Emergency Center (PEC). Physician’s recommendations were entered in the HIS to facilitate patient access to the ED. Non-life-threatening cases were served as regular OPD appointments. Physicians could order investigations, write/refill prescription/s, or refer patients to the OPD (routine/urgent). UCC’s OPD referrals were considered as triaged, with faster appointment booking. To ensure uniform high-quality service, telehealth protocols were developed for the triage and specialty physicians. (Figures 2 and 3). Appropriate documentation was entered in HIS in compliance with hospital documentation policies.

##### Quality Assurance

Despite that the UCC was created during an extra-ordinary pandemic, efforts were invested in maintaining the highest healthcare service quality, in line with the patient charter and Joint Commission International (JCI). Thorough patient identification before disclosing any medical information ensured patient privacy and that the physician was speaking to the correct patient/legal guardian. The only exception to this rigorous process was for calls requiring psychiatry/mental health services. As per the psychiatry department’s protocol, patient identification was not undertaken hence ensuring patient privacy and preventing stigma. Where medications/investigations were ordered, patient identification was undertaken prior to ordering. Physicians were assigned desks ≥ 2 m apart, with separators to avoid overhearing. Headphone and microphone sets were used during calls, and loudspeaker use during hotline consultation was not allowed.

Proper documentation of calls in HIS was audited daily in accordance with JCI standards. Visit notes included annotations confirming the virtual UCC consultation to assist the fast tracking of patients referred to OPD/ED/PEC. Patient waiting time until answered by the triage and specialty physicians was monitored to avoid unnecessary waiting; as well as audit of any missed calls, so that they could be called back during the same shift. All calls were recorded for service quality and to ensure high patient satisfaction.

#### Refining the UCC Service

The interactive dynamic model of the UCC operations was premised on analysis of the challenges encountered and workflow statistics, and changes were implemented accordingly as outlined below:

##### Working Hours

Initially, UCC working hours were 7am-10 pm, 7 days/week, however, as < 40 calls were received on Fridays, this schedule was changed to 6 days/week. During the holy month of Ramadan, calls decreased towards the end of the morning shift, leading to a further modification (2 shifts, 5 h each, morning 8am-1pm, evening 7pm-12am).

##### Interpretation

Qatar’s demography comprises 85% expatriates. Most people communicate in Arabic or English. However, once language barriers were identified during the calls, UCC assigned Indian, Malayalam and Urdu speaking Asian nurses for interpretation.

##### Document/Photo Sharing

Some patients wished to share photos with the attending physician (e.g., skin/oral lesions, old prescriptions, etc.). Hence, two dedicated mobile phones equipped with WhatsApp were allocated to the UCC. After any call, all files were immediately deleted from the mobile phones thus maintaining patient privacy.

##### Additional Medical Specialties

UCC initially included 11 specialties. Two weeks after its inauguration, based on patient demand, four more specialties were added (neurology, pain management, geriatric medicine, and oncology/hematology).

##### Additional Staff Subject to Call Volume

Based on call volume per specialty, additional attending physicians were recruited to avoid long waiting times and assist in returning any unattended calls. An example was the general medicine specialty, where 4 physicians instead of the traditional 2 were assigned per shift.

##### Medical Store Requests and Prescriptions

For chronic patients, store requests are needed for consumables (e.g., catheters, urine bags, etc.). Agreement with the HMC store allowed dispensing consumables to patients based on emailed store request copies from UCC. Likewise, for written prescriptions of selected medications not supplied by HMC pharmacy, MOPH issued changes allowing the use of an electronic copy of the prescription sent to patient via WhatsApp. In addition, agreement with HMC pharmacy bypassed the narcotic/antipsychotic prescriptions problem by daily written prescriptions sent from UCC to the narcotics pharmacy. Patients would then collect it after appropriate identification. HMC pharmacy also started free home delivery for all prescribed medications using the Qatar Post service. UCC physicians guided patients to this service.

##### Health Cards

Visitors to Qatar did not have health cards, and could not be served by UCC (cannot be registered into the system). The Patient Registration department granted access to UCC coordinators to create health card numbers for such patients permitting access to public healthcare.

##### UCC Encounter Code

Each patient visit to HMC is registered by clerks as an encounter. UCC, being a new service, had no encounter for the service. As UCC is physician-operated, the IT department generated a UCC-specific encounter code, created by triage physicians during the call, ensuring appropriate patient documentation/registration.

## Results

### Physician Characteristics

The 15 triage physicians were new medical graduates with 1–5 years experience, and age range 28–32 years. The 150 specialty physicians were senior staff with 6-45year experience, and age range 30-75years.

### Caller Characteristics

Qatari nationals comprised about a third of the callers, patients’ median age was 46 years, and males comprised 50.7% of callers (Table 1). Callers’ sex differed by specialty (females 100% in obstetrics/gynecology, 13.3% in urology) (Table 2). Most calls (89%) were in Arabic or English language, with interpretation required for only 11% of calls.

**Table 1:** Selected characteristics of patients calling UCC during the first wave

Characteristic	Value
Nationality	
Qatari nationals	21,334 (38%)
Arab expatriates	19,088 (34%)
Non-Arab expatriates	15,718 (28%)
Gender	
Males	28,463 (50.7%)
Females	27,677 (49.3%)
Age (years)	Median 46
	Range (Newborn-101)
Interpretation required	6535 (10.85%)

Descriptions of patients calling for psychiatry/ mental health services are not included as per departmental policy

**Table 2:** UCC callers’ age and gender by specialty

Characteristic	Urology	ENT	Gynae	Peds	Surg	Medicine	Cardio	Neuro	Ortho	Derma	Pain	Geriat	Dental	Onco/Hema
Age, years														
Median Range	36 (12–95)	36 (12–95)	35 (10–84)	6 (0–14)	41 (13–93)	46 (14–59)	54 (12–109)	42 (10–98)	41 (12–98)	44 (1–96)	36 (16–97)	66 (60–101)	23 (12–74)	41 (16–86)
Males n (%)	2682 (86.7)	1276 (70)	0 (0)	1289 (53.6)	795 (41.2)	7381 (49.8)	3787 (72.1)	1388 (52.5)	1935 (48.7)	1605 (50.2)	883 (64.8)	3154 (43.7)	844 (45.5)	370 (28.6)
Females n (%)	410 (13.3)	547 (30)	5221 (100)	1114 (46.4)	1135 (58.8)	7440 (50.2)	1469 (27.9)	1255 (47.5)	2035 (51.3)	1592 (49.8)	480 (35.2)	4055 (56.3)	1013 (54.5)	989 (71.4)

Cell values represent N (%) except when the range is presented ENT: ear, nose, throat; Gynae: gynecology; Peds: Pediatric; Surg: Surgery; Cardio: cardiology; Neuro: neurology; Ortho: orthopedics; Derma: dermatology; Pain: Pain management; Geriat: Geriatrics; Onco/Hema: oncology/hematology. Descriptions of patients calling for psychiatry/ mental health services are not included as per departmental policy

### Call Characteristics

The response rate varied from 89 to 100%, with different monthly rates per specialty (Table 3). The maximum total daily calls peaked at 1670 calls on June 14, 2020. Call volumes were the highest from 9 am-2 pm. Median call waiting time before the triage physician answered was 1.5 min (range 1–12 min). As for the different specialties, median call waiting was 3.5 min (range 1.5–24 min).

**Table 3:** Response rates to inbound calls by specialty and month of lockdown

Month		Urology	ENT	Gynae	Peds	Surgery	Medicine	Cardio	Neuro	Ortho	Derma	Pain	Geriat	Dental	Onc/Hem	Total
April	Incoming calls	457	241	679	441	312	2105	837	398	550	476	156	399	238	231	7520
	Answered	423	240	615	441	312	1975	779	345	541	448	139	385	238	231	7112
	Response rate	93%	99%	91%	100%	100%	94%	93%	87%	98%	94%	89%	97%	100%	100%	96%
May	Incoming calls	539	321	1010	469	331	2569	948	400	704	474	232	1250	296	309	9852
	Answered	519	316	944	469	323	2456	914	354	677	455	210	1238	296	309	9480
	Response rate	96%	99%	93%	100%	98%	96%	96%	89%	96%	96%	91%	99%	100%	100%	96%
June	Incoming calls	838	570	1388	655	474	4249	1328	601	1117	837	299	1893	441	324	15,014
	Answered	807	570	1357	655	446	4093	1308	568	1097	803	282	1878	441	324	14,629
	Response rate	96%	100%	98%	100%	94%	96%	98%	94%	98%	96%	94%	99%	100%	100%	97%
July	Incoming calls	622	360	1210	453	464	3432	1154	595	869	811	354	1844	486	268	12,922
	Answered	597	340	1094	453	246	3338	1067	531	850	752	318	1774	486	268	12,114
	Response rate	96%	94%	90%	100%	53%	97%	92%	89%	98%	93%	90%	96%	100%	100%	94%
August	Incoming calls	636	331	934	385	349	2466	989	649	730	599	322	1822	396	226	10,832
	Answered	612	331	902	385	335	2414	942	614	716	564	300	1780	396	226	10,517
	Response rate	96%	100%	97%	100%	96%	98%	95%	95%	98%	94%	93%	98%	100%	100%	96%
Overall Period	Incoming calls	3092	1823	5221	2403	1930	14,821	5256	2643	3970	3197	1363	7208	1857	1358	56,140^*a*^
	Answered	2958	1797	4913	2403	1662	14,276	5008	2412	3881	3022	1249	7055	1857	1358	53,851
	Response rate	96%	99%	94%	100%	86.1%	96%	95%	91%	98%	95%	92%	98%	100%	100%	96%

ENT: ear, nose, throat; Gynae: gynaecology; Peds: pediatrics; Cardio: cardiology; Neuro: neurology; Ortho: orthopedics; Derma: dermatology; Pain: Pain management; Geriat: Geriatrics; Onc/Hem: oncology/hematology; ^*a*^ total does not add to 60,229 due to exclusion of patients calling for the psychiatry/mental health services as per the departmental protocol

### Specialty Characteristics

**Fig. 4:**
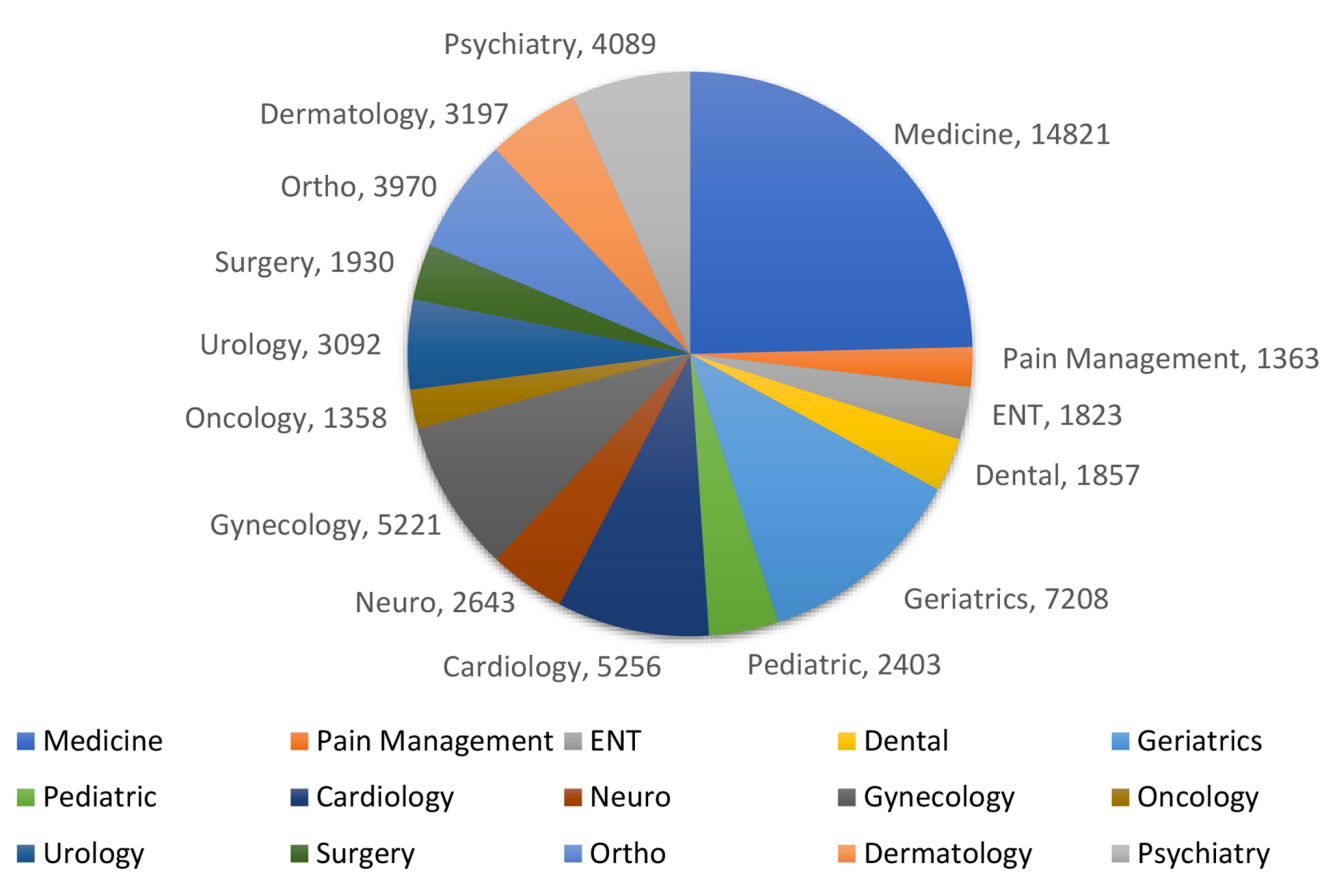
Total call distribution by specialty across 5 months of the first COVID-19 lockdown

**Fig. 5:**
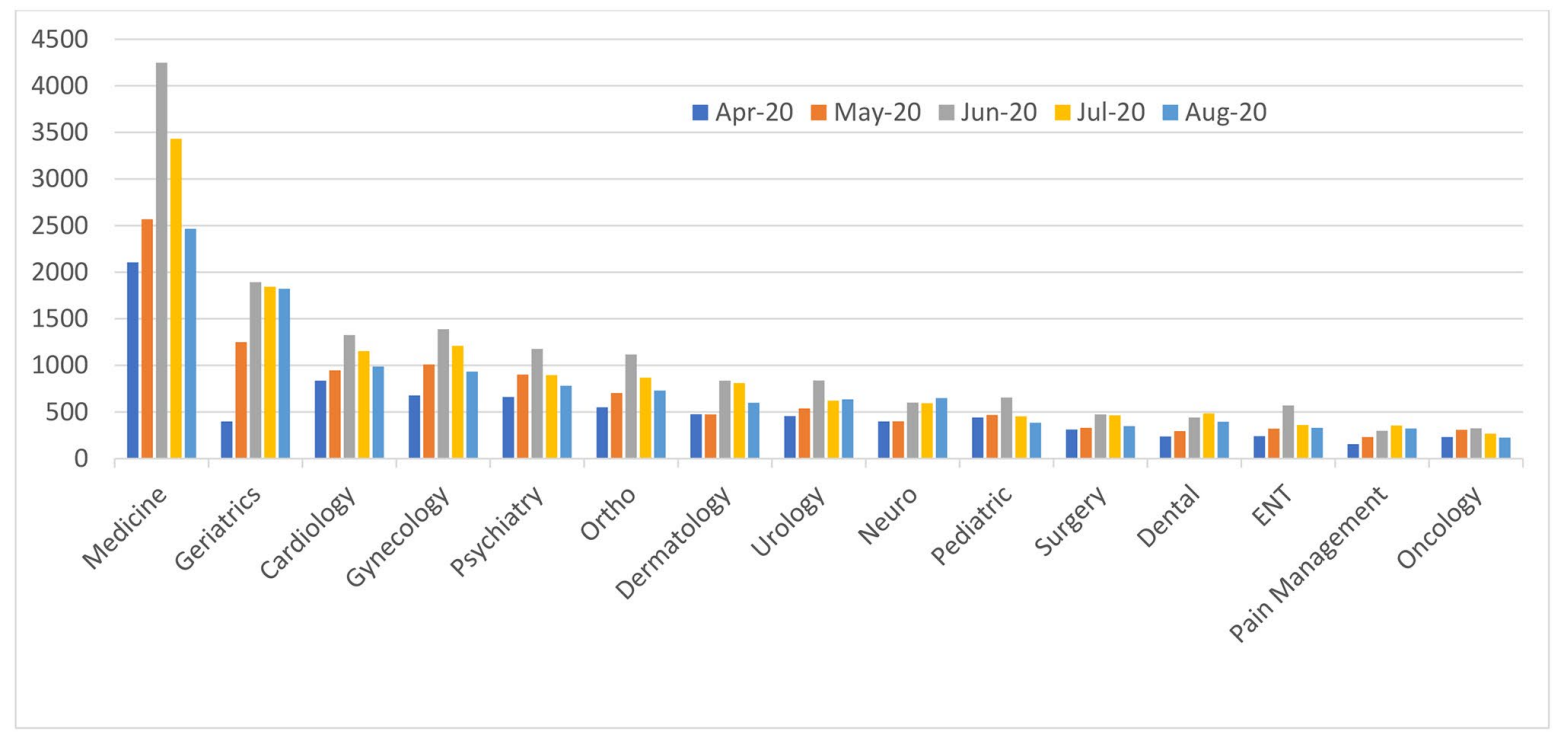
Call distribution by specialty across 5 months of the first COVID-19 lockdown. Ortho: Orthopedics; Neuro: Neurology; ENT: Ear, Nose and Throat

**Fig. 6:**
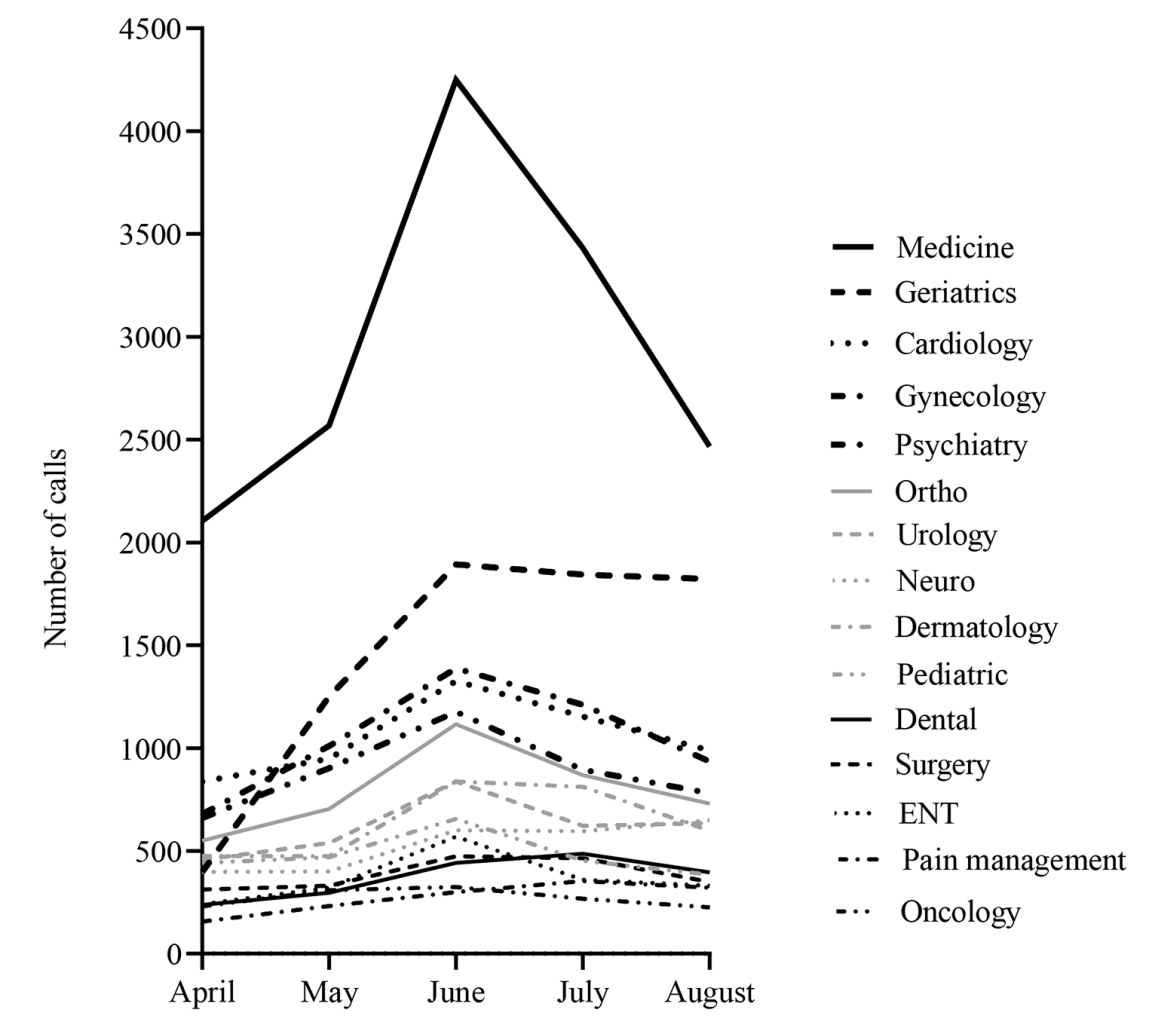
Number of calls by month across specialties

Fifteen specialties were covered by UCC. During the study period, UCC received 60,229 calls (average of 394 calls/day). After initial telephone triage, callers were most often directed to internal medicine (24.61%), geriatrics (11.97%), cardiology (8.72%), gynecology (8.66%), psychiatry (6.79%), orthopedics (6.59%), dermatology (5.30%), urology (5.13%), neurology (4.29%), and pediatrics (3.99%); while other specialties included general surgery, ENT and dental (each 3%); and around 2% for each of pain management and oncology/hematology (Fig. 4). The call volume and ranking of the specialties based on total monthly calls differed by month (Fig. 5). Figure 6 shows the fluctuation of calls with time for the various specialties, illustrating a spike increase during June due to the second wave of COVID-19.

### Outcomes

**Fig. 7:**
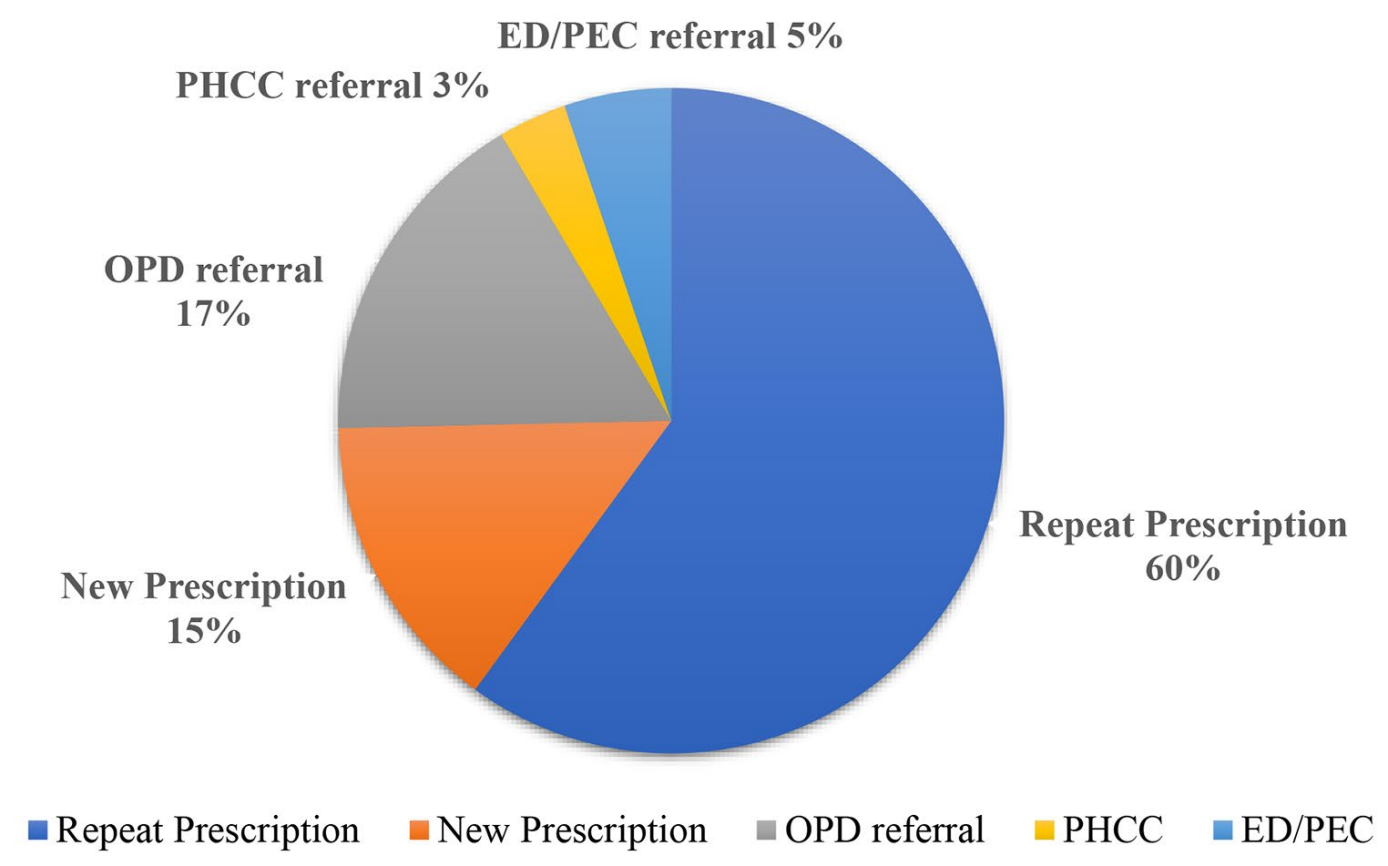
Outcome of the UCC calls for all specialties. PHCC: primary healthcare center; ED: emergency department; PEC, pediatric emergency center; OPD: out-patient department

Analysis by outcome of the consultation showed that the majority of calls were truly non-emergency, with repeating prescriptions being the most provided service (60% of calls). However, 5% of calls were true emergencies were the patient was advised to go to ED/PEC (5%) immediately (Fig. 7).

Table 4 depicts the outcome of the calls by specialty. The highest volumes of medication refills were for medicine and geriatrics specialties (51.19%), followed by neurology and orthopedics. As for new prescriptions, the most common were medicine and dermatology (42.04%), followed by obstetrics-gynecology and ENT. In terms of referrals, OPD referrals were most common from orthopedics (33.57%) followed by medicine and surgery (24.58%); referrals to PHCC were most common from dentistry and medicine (47.63%) followed by obstetrics-gynecology. Referrals to emergency department were most common from obstetrics-gynecology, pediatrics, medicine (54.41%) followed by cardiology and surgery.

**Table 4:** Outcome of calls by specialty

Specialty	Outcome				
	Medication		Referral		
	Refill	New Prescription	OPD	PHCC	ED
Medicine	7855 (31.85)	1353 (22.85)	979 (14.34)	489 (36.06)	358 (17.06)
Surgery	273 (1.11)	385 (6.50)	699 (10.24)	28 (2.06)	238 (11.34)
Neurology	1576 (6.39)	170 (2.87)	134 (1.96)	5 (0.37)	101 (4.81)
Urology	1264 (5.12)	476 (8.04)	510 (7.47)	34 (2.51)	166 (7.91)
Dentistry	45 (0.18)	109 (1.84)	352 (5.16)	523 (38.57)	40 (1.91)
Dermatology	1229 (4.98)	1136 (19.19)	438 (6.42)	1 (0.07)	7 (0.33)
Obs-gynae	812 (3.29)	843 (14.24)	531 (7.78)	175 (12.91)	393 (18.72)
Cardiology	3343 (13.55)	187 (3.16)	165 (2.42)	18 (1.33)	228 (10.86)
Pediatrics	428 (1.74)	534 (9.02)	79 (1.16)	21 (1.55)	391 (18.63)
Orthopedics	1620 (6.57)	4 (0.07)	2291 (33.57)	0 (0)	6 (0.29)
ENT	445 (1.8)	489 (8.26)	387 (5.67)	30 (2.21)	131 (6.24)
Pain Management	1005 (4.07)	96 (1.62)	50 (0.73)	1 (0.07)	4 (0.19)
Geriatrics	4769 (19.34)	138 (2.33)	210 (3.08)	31 (2.29)	36 (1.72)

Cell values represent frequency (%); Obs-gynae: obstetrics-gynecology; ENT: ear, nose, throat; OPD: outpatient department; PHCC: primary health care centre; ED: emergency department

## Discussion

In this service evaluation report, we described the feasibility, organization and effectiveness of a large-scale physician-staffed hotline providing care and disseminating information to a country-wide general population and patients requiring urgent care amidst a global pandemic. The main findings were that, during the five months under examination, about 60,000 patients called the hotline, were triaged and immediately connected to physicians specialized in 15 different medical and surgical specialties. As the volume of calls to the UCC hotline surged, the ability of the UCC’s 150 triage physicians manning the hotline to answer calls as they came in varied from 89 to 100%. For 75% of callers, repeat and new prescriptions (60% and 15% of calls respectively) were the reason for the call and were provided, thus maintaining a seamless continuity of care with no additional exposure risk to COVID-19 virus in terms of an uninterrupted flow of critically required medications and crucial essential treatments to patients.

As regards the technology, perhaps the key to the success of hotlines is their relative simplicity compared to other modes of remote telemedicine. We agree that telephone hotlines entail modest technological competencies, can be instigated swiftly, and are available to those who have no internet access, particularly when facilities with public internet e.g., libraries/ wi-fi cafes are inaccessible due to the lockdown [30]. Indeed, UCC’s hotline was set up in seven days as the technology required was feasible and swiftly provided a synchronous and efficient real-time interactive health service. Others have observed that telephone calls have comparable patient health outcomes as video-based appointments [47]. As illustrated in setting up the UCC in Qatar, early during the pandemic, only the audioconferencing approach was initially available, hence remote consultations were limited to telephone consultations [48].

In terms of manpower and competencies, the physicians who manned the UCC hotline comprised generalists for the triage who then transferred the call accordingly to specialist and consultant physicians, surgeons and dentists selected to reflect the abilities and expertise required for the task. Others have highlighted that the workforce could be selected for their expertise [49]; and telephone advice depends on an appropriately skilled workforce who are accessible to sort out and settle calls over the phone, with attention to patients’ emotional and medical needs [50]. Most studies reported hotline service/s that were manned by non-physicians e.g., nurses, volunteers, counselors, psychologists, social service agencies or operators [2, 13, 15, 32, 35, 39]. The competencies of such a variety of operators would cover, to an extent, some ‘hard’ skills e.g., basic knowledge and intervention skills, as well as a wider range of ‘soft’ skills e.g., “ability to build a relationship with the callers effectively,” “quickly focus on the major complaint of the callers and form the primary intervention plan,” and “ability to identify and respond to emergencies and nuisance calls” [40]. Nevertheless, such level of competencies would be different from those of a fully trained physician conducting the triage at UCC or specialized physicians by specialty conducting the consultations as seen in the current report. Physician-staffed hotlines are very scarce in the literature, mostly limited to COVID-19 service, e.g. [30]. In this respect, Qatar’s UCC is truly unique as it was physician-staffed and serviced all medical and surgical specialties. Research is required to establish the necessary skill level and education, and the decision support strategies to ensure uniform, appropriate and safe care via telephone advice [51].

As a health service model during a pandemic, the present innovative UCC model confirms that robust, comprehensive, and hospital-integrated hotline-based telehealth is a viable health service model during the acute phase of a pandemic. The timely initiation of UCC, and the subsequent adjustments and refinements to its operations as highlighted above, provide support that during the period of practice reconfiguration world-wide, the renewed interest for hotline telemedicine led to promising service delivery changes [52]. Across the volume of calls received by UCC, the mean waiting time for a call to be answered was 1-1.5 min, which was excellent. In addition, after the call was answered and connected to the required specialty, about 75% were resolved successfully (only ≈ 25% of calls were referred). These findings agree with that telephone advice can deliver appropriate and timely clinical response for some patients, settling low acuity calls [49]; and, a systematic review of telephone advice observed that clinician advice and disposition assigned were both safe and appropriate [51]. UCC was successful in limiting face-to-face visits to the hospital, as the service managed the complaints of about 45,000 patients remotely, significantly reducing the number of patients who would have otherwise physically visited various HMC facilities. Others noted similar findings [4].

In terms of referrals, 25% of calls were referred, comprising mainly to outpatient departments (17%), while referrals to emergency departments and PHCC were minimal (5% and 3% respectively). This highlights that even when referrals were undertaken, only 5% required the urgency and skills available in an emergency department. This is critical, given that many emergency departments were dealing with COVID-19 cases. Our observations support findings reported elsewhere: on the one hand, patients need to avoid delaying necessary medical care during the pandemic, especially in emergency situations [53]; on the other hand, individuals are advised to avoid unnecessary healthcare use to reduce transmission of the virus and ensure that hospital capacity can accommodate surges in COVID-19 cases [54].

As a future post-pandemic health service model, the UCC hotline model has promise and future implications. The savings in healthcare provider and patient time, effort, transport and pressure on the healthcare system that a robustly-set and operated hotline offers definitely need to be considered after the pandemic has subsided. Hotlines will most probably be a permanent ‘modus operandi’ for the future that is both cost and clinically effective. The longer-term prospect of hotlines telemedicine is likely to persist after the pandemic’s acute stages [52].

The current report has some limitations. Missing data are not uncommon in retrospective inquiries of data routinely collected as part of service audit. Likewise, others have raised some concerns about the consistency of data reporting for calls resolved over the phone [55], and the current study is no exception. Although research exists on the demographic characteristics of e.g., persons calling for dental care [4], the current report, akin to other reports that employed administrative data about calls, is not in a position to remark on the features of the callers who made the calls [15]. A breakdown of emergency conditions detected and referred to the emergency department by the hotline would have been useful to provide an indication of the impact of the hotline in the detection of emergency cases and hence saving lives. However, the data to undertake this task is unavailable.

Notwithstanding, the current report has many strengths. It assessed all calls to a new broad-scope service provided to a whole country, covering all specialties. We analyzed routinely collected information on the total calls to a national hotline covering the whole nation, where other studies examined only city-wide [17] or state-wide [35] initiatives. Hence it is wide scale and the calls captured are representative of the demographically and ethnically diverse population in Qatar as HMC is the main public tertiary care provider. Calls comprised nationals and the breadth of the multinational populations that are resident in Qatar with different ethnic and genetic backgrounds, rendering generalization of the findings feasible. In addition, the report appraised all calls, and research employing the total population of calls for a hotline service that serves all specialties of healthcare, as we undertook, is not common, e.g., [15]. Likewise, we evaluated a service that encompassed all healthcare specialties, where other studies were only COVID-19-specific or appraised single condition/disease or individual healthcare specialties, e.g., [13, 31, 33]. The report also provides information across the complete period of the first lockdown, furnishing a broad view across five months.

## Conclusion

The high call volume highlights the demand on and the utility of the UCC in bridging the unavoidable gaps that emerged during the pandemic, thus ensuring a smooth transition and continuity of care. The hotline data underline the importance of having a consistent, trustworthy and specialized source of care and information across all healthcare specialties for the public and patients when other services are unavailable. With adequate resources and much coordination, the service setup as well as its operations ran smoothly, and the triage physicians as well as the physicians in the different specializations were able to deliver UCC’s objectives. The findings provide valuable insights on the nature of the process and key challenges for other similar settings instigating hotline services.

## Appendix

**Table 5:** Summary Table

What was already known on the topic:	What this study added to our knowledge:
The importance of hotlines in the delivery of healthcare remotely is recognized Healthcare hotline service/s worldwide are mostly operated by non-physicians. Healthcare hotline service/s mostly service a single medical condition, rather than the broader spectrum of medical and surgical specialties.	Physician-operated healthcare hotline service/s hotline can contribute to reducing outpatient department walk-in, particularly in crises or pandemic situations, for urgent non-life-threatening conditions maintaining the continuity of care while decreasing the risk of viral infection Physician-operated healthcare hotline service/s hotline can detect life-threatening and emergency conditions and refer such cases to appropriate emergency services and departments thus maintaining the safety of critically ill cases The setting up and smooth functioning of hotlines servicing a spectrum of medical and surgical specialties require a complex web of interactions, and intersectoral and interdepartmental collaboration between various ministries, hospitals and institutes, as well as departments, and specialties
